# High-velocity nasal insufflation in dogs with left-sided congestive heart failure unresponsive to traditional oxygen therapy: a retrospective case series

**DOI:** 10.3389/fvets.2025.1562633

**Published:** 2025-05-09

**Authors:** Bailey Lane, Rebecca A. Walton, April E. Blong, Meredith ‘t Hoen, Melissa A. Tropf, Jessica L. Ward, Allison K. Masters

**Affiliations:** ^1^Department of Veterinary Clinical Sciences, Iowa State University, Ames, IA, United States; ^2^VCA West Los Angeles, Los Angeles, CA, United States

**Keywords:** congestive heart failure, canine, high-velocity nasal insufflation, HVNI, high flow

## Abstract

**Objective:**

To describe high–velocity nasal insufflation (HVNI) for managing dogs with left–sided congestive heart failure (L–CHF) failing traditional oxygen therapy (TOT). To secondarily evaluate complications based on retrospective evaluation of the record of HVNI and survival to discharge.

**Design:**

Retrospective case series from a university teaching hospital between August 2019 and October 2021.

**Animals:**

Twelve dogs diagnosed with L-CHF and managed with HVNI.

**Measurements and main results:**

Medical records were retrospectively reviewed for signalment, point-of-care diagnostics, and HVNI information. Nine dogs were diagnosed with myxomatous mitral valve disease, and three dogs were diagnosed with dilated cardiomyopathy. All dogs in this study required HVNI after failing TOT. Dogs were treated with HVNI for a median of 14 h (range 2–22 h). HVNI was successfully discontinued in 10 dogs (83%), all of which survived to discharge. Two dogs on HVNI were humanely euthanized, both of which were diagnosed with Stage D refractory CHF. No major complications of HVNI were noted in any dogs.

**Conclusion:**

HVNI is a potential method of escalating oxygen support for dogs in L-CHF who fail TOT. In this case series, all dogs in which HVNI was successfully discontinued survived to discharge.

## Introduction

Left sided congestive heart failure (L-CHF) secondary to primary cardiac disease is a common cause of respiratory distress in dogs (1). The development of L-CHF results in hypoxemia due to pulmonary edema, and emergency treatment includes sedation, diuretic administration, and oxygen supplementation.

Oxygen administration to treat hypoxemia in dogs with L-CHF is most often provided via traditional oxygen therapy (TOT) using flow-by oxygen, masks, nasal cannulas, or oxygen cages (2). For many patients, TOT and targeted cardiac therapies, including diuretics and positive inotropes, are sufficient to address hypoxemia and clinical evidence of respiratory distress; however, some patients with L-CHF require more aggressive oxygen support. Mechanical ventilation (MV) has been successfully used in dogs with L-CHF failing TOT, with a survival to discharge rate of 77% (3). While the survival to discharge rate for dogs undergoing MV secondary to L-CHF is good, MV can be associated with a wide range of complications including but not limited to ventilator associated pneumonia, ventilator associated lung injury, and significant financial burden (3).

In people, high velocity nasal insufflation (HVNI) is used to bridge the gap between TOT and MV (4). HVNI has been evaluated in dogs with hypoxemia of various etiologies that have failed TOT, demonstrating improvement in partial pressure of oxygen and respiratory status; however, HVNI has not been specifically evaluated in dogs with L-CHF (4). The goal of this study was to describe the use of HVNI in dogs diagnosed with L-CHF that failed TOT. Secondary objectives were to evaluate complications of HVNI use and report survival to discharge of dogs with L-CHF managed with HVNI.

## Materials and methods

The electronic medical records from the Lloyd Veterinary Medical Center at Iowa State University were searched for all patients treated with HVNI between August 2019–October 2021. Dogs were included in the study if they were diagnosed with L-CHF based on the clinical finding of respiratory distress, thoracic point of care ultrasound that identified B-lines, and an echocardiogram consistent with left-sided heart disease performed by a board-certified cardiologist or cardiology resident. Specific locations of B-lines were not available based on retrospective review of records. Cases were excluded if a non-cardiac indication for HVNI was noted or if an echocardiogram was not performed.

The following information was collected for each case: signalment, body weight, and cardiac disease diagnosis; diagnostic imaging results (echocardiogram and thoracic radiography); treatment prior to HVNI; HVNI settings and duration; complications of HVNI, including air-leak syndrome or intolerance of oxygen delivery or nasal cannulas, based on dislodgement or need for additional sedation; and survival to discharge.

## Results

Seventy-five dogs undergoing HVNI during the study period were identified. Sixty-three dogs were excluded because HVNI was utilized for non-cardiac causes of respiratory distress. Twelve dogs met the inclusion criteria. Six dogs were castrated males, five were spayed females and one was an intact male. Breeds in this study included Miniature Dachshund (2), mixed breed dogs (2), and one of each of the following: Japanese Chin, Chinese Crested, Cavalier King Charles spaniel, Lhasa Apso, Doberman Pinscher, Chihuahua, Yorkshire terrier, and Chow Chow. The median weight was 12.5 kg (range 1.82–32.8 kg). The median age was 11.5 years (range 8–16 years).

All dogs had an echocardiogram, performed by a cardiology resident or board-certified cardiologist, documenting acquired heart disease. Ten of the dogs had an echocardiogram performed following hospital admission and the remaining two dogs had prior echocardiography at the authors’ institution, with a known diagnosis of Stage C L-CHF. The primary acquired heart disease diagnosis was myxomatous mitral value disease (MMVD) in nine dogs and dilated cardiomyopathy (DCM) in three dogs. Seven dogs (58.3%) were experiencing their first episode of L-CHF, four dogs (33.3%) were experiencing a second episode of L-CHF, and one dog (8.3%) was experiencing a fourth episode of L-CHF. Based on diuretic dose at the time of HVNI, 10 dogs were classified at Stage C L-CHF while two dogs were classified at Stage D due to lack of response to conventional medical management (5). Nine of the 12 dogs also had thoracic radiographs, interpreted by a board-certified radiologist or cardiologist and confirming a diagnosis of L-CHF, performed within the first 12 h of hospitalization.

All dogs undergoing HVNI received TOT prior to initiation of HVNI. One dog was hospitalized for polyuria and hypoalbuminemia at the time of onset of congestive heart failure. For this dog, length of TOT was calculated from the time of onset of CHF symptoms and initiation of oxygen and diuretic therapy to the time of transition to HVNI. In three dogs, due to the severity of respiratory distress on presentation, the decision to institute HVNI was made immediately and these dogs received TOT for 45 min or less while HVNI was being set up and owner permission to continue care was obtained. The remaining nine dogs were administered TOT for an average of 5.25 h (range 1.08–17.25 h) before escalation to HVNI. The two dogs in Stage D L-CHF were escalated to HVNI after 45 min and 90 min. Failure of TOT was determined by attending clinician discretion, with one or more of the following criteria noted before escalation: increased work of breathing, impending respiratory fatigue, or hypoxemia as noted by PaO_2_ of less than 60 mmHg on an arterial blood gas or by a peripheral oxygen saturation of less than 90%.

Once the decision to start HVNI (DRE Volumax from Avante Health Solutions, Louisville, KY) was made, all dogs were instrumented with nasal cannulas, ensuring the cannula occluded < 50% of the nares. Butorphanol was administered at the time of cannula placement in 11/12 dogs at a median dose of 0.2 mg/kg (range 0.1–0.3 mg/kg). Adult and pediatric circuits are available for HVNI; dogs weighing less than 12 kg were placed on pediatric circuits and dogs weighing more than 12 kg were placed on adult circuits. The median flow rate at the time of initiation was 1.1 L/kg/min (range 0.7–2.2 L/kg/min), the median fraction of inspired oxygen (FiO_2_) was 1.0 (range 0.5–1.0), and the temperature was set to 37°C for all cases. Dogs were maintained on HVNI for a median of 14 h (range 2–22 h).

No major complications of HVNI were noted in any dog during the study, including clinically significant pneumomediastinum, pneumothorax, or subcutaneous emphysema. Three of the twelve dogs had thoracic radiographs performed at variable timepoints following initiation of HVNI, with no radiographic evidence of pneumomediastinum, pneumothorax or subcutaneous emphysema. In general, nasal cannulas and HVNI were well tolerated; however, minor irritation was noted in eight dogs, and these complications were addressed with an Elizabethan collar (four dogs), topical anesthesia such as proparacaine drops in the nares (precise number of dogs unknown due to inconsistent record keeping), trazodone (three dogs, dose range 2.67–3.75 mg/kg as needed), and/or systemic sedation using butorphanol. Eight dogs received intravenous butorphanol (dose range 0.2–0.4 mg/kg) on an as needed basis; one of these dogs was transitioned to a constant rate infusion of butorphanol (0.2–0.4 mg/kg/h) due to persistent patient attempts remove the cannula.

Ten dogs (83%) were successfully discontinued from HVNI. Initial settings were determined based on pulse oximetry, respiratory rate, accessory muscle excursions and/or evidence of respiratory acidosis. Venous blood gas analysis was performed in two patients immediately prior to the initiation of HVNI. In both patients, a respiratory acidosis was noted (CO_2_ 59.6 mmHg and 52.3 mmHg [reference range: 29–42 mmHg]). If one or more of these parameters improved, the FiO_2_ was decreased in 0.05–0.1 increments as rapidly as possible to minimize the risk of oxygen toxicity. Of these 10 dogs, seven required de-escalation of oxygen administration to TOT prior to transitioning to room air, whereas three dogs were immediately transitioned to room air. De-escalation methods were based on patient clinical picture and HVNI settings at time of discontinuation of HVNI. The two dogs that were not discontinued from HVNI were humanely euthanized while receiving HVNI. One dog received 2 h of HVNI, and one dog received 4 h of HVNI. One dog with stage D DCM was humanely euthanized due to concern for long-term prognosis, and one dog with stage D MMVD was euthanized due to the development of an acute kidney injury. Euthanasia was due to owner preference or non-cardiac related reasons in both cases; no dogs were euthanized due to progressive respiratory failure or hypoxemia despite HVNI. No dog in this study died spontaneously while on HVNI or during their hospitalization. Nine dogs were discharged with furosemide at a median dose of 3.8 mg/kg/day (range 1.26–8.1 mg/kg/day), and one dog was discharged with torsemide at 0.34 mg/kg/day. All dogs were discharged with pimobendan at a median dose of 0.65 mg/kg/day (range 0.32–1 mg/kg/day). Five dogs were discharged with angiotensin converting enzyme inhibitors at a median dose of 0.48 mg/kg twice daily (range 0.39–0.65 mg/kg). Other medications prescribed in some cases included oral potassium supplementation, diltiazem, digoxin, spironolactone, sotalol, clopidogrel and hydrocodone. The median length of hospitalization was 34 h (range 18–92 h).

## Discussion

The prognosis for dogs treated with HVNI for L-CHF in the present study was good, with 10/12 (83%) dogs surviving to discharge. Acute treatment and stabilization of L-CHF relies on oxygen supplementation. Traditional oxygen therapy involves low-flow systems that deliver oxygen at a flow rate lower than patient ventilatory requirements, resulting in dilution of inspired oxygen concentration (2). Negative patient effects of TOT result from the delivery of cold, dry air, which can cause airway desiccation, patient discomfort, and intolerance of oxygen delivery, as well mucociliary dysfunction, bronchospasm and airway inflammation (1, 2, 6). In a previous retrospective study of dogs and cats in L-CHF, 59% of animals received TOT with a median time in oxygen of 24 h with a survival to discharge of 80% (7). Dogs failing TOT require an escalation in treatment, which has historically meant MV. Dogs in L-CHF that require escalation to MV have a prognosis for survival to discharge with MV of ranging from 54 to 77% (3, 8). Despite these high survival rates, MV is associated with significant financial burden and a high incidence of complications. Potential complications associated with MV for L-CHF include ventilator associated pneumonia, acute respiratory distress syndrome, and the development of pneumothorax (8). Therefore, the ability to escalate care without progressing to MV would be of benefit to some dogs with L-CHF. High velocity nasal insufflation provides gas at a rate that matches minute ventilation, delivers a humidified gas mixture, and provides an FiO_2_ up to 1.0 (2).

High velocity nasal insufflation has been utilized in the acute management of CHF in people due to its mechanisms of oxygen support and hemodynamic effects that aid in reducing pulmonary edema through a reduction in cardiac preload and afterload (9). High velocity nasal insufflation increases intrathoracic pressure, which may result in inspiratory collapse of the cranial vena cava, resulting in decreased preload (10). The provision of positive end expiratory pressure (PEEP) and reduced sympathetic nervous system tone that occurs with the amelioration of hypoxia can both work to reduce afterload (11). High velocity nasal insufflation has also been described in dogs with acute hypoxemic respiratory failure with noted increases in PaO_2_ and decreases in dyspnea scores with an overall survival to discharge of 45% in one study (12). High velocity nasal insufflation therapy has not been specifically evaluated in dogs with L-CHF, and no definitive guidelines are established in dogs regarding HVNI indications and settings. The aim of this study was to describe the treatment and outcome of dogs with L-CHF treated with HVNI.

High flow nasal oxygen (HFNO) is an umbrella term that provides oxygen-rich, heated, humidified gas to the patient via nasal cannulas (9). Types of HFNO include high flow nasal cannula (HFNC) and high velocity nasal insufflation (HVNI). These types of high flow nasal oxygen administration differ in that HVNI delivers gas at a much higher velocity compared to HFNC at similar flow rates. Velocity is determined by the cross-sectional area and flow rate; therefore, the small diameter of the HVNI nose piece provides faster gas delivery at lower flow rates (13). This means that HFNC flow rates of 60 L/min are similar to HVNI flow rates of 40 L/min (13). High velocity nasal insufflation administers a customizable FiO_2_, up to 1.0, at high velocity, which provides more oxygen support and decrease the work of breathing compared to TOT (14). High velocity nasal insufflation systems utilize variable-sized nasal cannulas to achieve high velocity, allowing for rates up to 40 L/min in comparison to TOT rates of 0.1–0.4 L/kg/min while also warming and humidifying air (14). No maximal L/kg/min flow rate has been established for HVNI. The benefits include prevention of airway desiccation and epithelial injury, decreased work of breathing, and provision of PEEP.

General recommendations for HVNI in veterinary medicine include initial set up including an FiO_2_ of 1.0 while stabilization occurs, along with flow rates of 1–2 L/kg/min, which has been noted to be well tolerated and aid in reduction in the need of MV in human pediatric patients (2, 15). In this study, the median FiO_2_ at the time of initiation was 1.0 and median flow rate was 1.1 L/kg/min, similar to previous studies. Previous descriptions of HVNI in dogs include the need for sedation to facilitate cannula placement, similar to the dogs in this study (2, 16). Most dogs received butorphanol around the time of nasal cannula placement, but due to the retrospective nature of this study it is unknown whether the butorphanol was administered primarily to facilitate cannula placement or for the clinical signs of respiratory distress. Regardless, sedation in addition to butorphanol was not required in any dog for cannula placement. During hospitalization, eight dogs received butorphanol or trazodone, but whether this was solely related to patient intolerance of the HVNI system or other factors could not be ascertained.

An established protocol for de-escalation of HVNI is not available in veterinary medicine and human guidelines vary (2). General considerations include improvement of the primary condition, decreased device settings including FiO_2_ and flow rate, and improved work of breathing (2). In this study, 10 dogs were successfully weaned from HVNI, and all of those dogs survived to discharge. Seven of the dogs successfully weaned from HVNI were transitioned to TOT before discharge while three dogs had HVNI discontinued with immediate transition to room air based on clinical picture and HVNI settings at time of discontinuation. The protocol for titration and de-escalation of HVNI therapy was based on clinician preference. A consensus statement for human neonates recommends titration of FiO_2_ to less than 0.3 before decreasing flow rates (4, 17). This consensus statement suggests that flow can be reduced when respiratory rates and work of breathing are stable for more than 12–24 h and discontinuation to TOT can be considered when the flow rate is less than or equal to 1 liter/kg/min.

Complications of HVNI are rarely reported in dogs and are typically minor, including irritation associated with nasal cannula placement and aerophagia (10, 16). Hypercapnia, among the most commonly reported complications of HVNI in dogs, is suspected to be due to sedation and increased resistance to expiration associated with the high inspiratory flow rates (2, 16). However, washout of nasopharyngeal dead space during HVNI decreases rebreathing of carbon dioxide and may play a mitigating role in the development of hypercapnia (18). More serious complications such as the development of a pneumothorax have been reported in people and dogs (13, 14). Other complications of HVNI are reported in people, including facial trauma, abdominal distention, aspiration and barotrauma, but are rare or not documented in veterinary medicine (2). In this study, complications were mild and included nasal cannula intolerance in some dogs; however, this was easily managed with topical analgesia and sedation. Hypercapnia was not specifically evaluated in this study.

Limitations of this study include the retrospective nature and small sample size. Additionally, there was no standardization of treatment, including the criteria for failing TOT and indication for HVNI. Dogs may have improved predominantly with therapeutic interventions, including the administration of diuretics, which may have impacted outcome. The decision to euthanize two dogs was made in conjunction with owner preference considering long-term prognosis, which affected outcome data.

In conclusion, in this small retrospective case series, the use of HVNI in dogs with L-CHF was associated with a high survival to discharge and low risk of complications. The survival to discharge in this study was 83%, which is higher than previously reported survival to discharge in dogs undergoing MV for L-CHF and higher than previously reported survival to discharge for dogs undergoing HVNI. HVNI is a reasonable consideration for dogs with L-CHF that fail TOT. Future directions include the prospective evaluation of HVNI in dogs with L-CHF with standardization of protocols.

## Data Availability

The original contributions presented in the study are included in the article/supplementary material, further inquiries can be directed to the corresponding author.
